# The development of a digital intelligence quotient scale: A new measuring instrument for primary school students in China

**DOI:** 10.1016/j.heliyon.2024.e36437

**Published:** 2024-08-16

**Authors:** Jing Li, Soon-Yew Ju, Caixia Zhu, Ying Yuan, Min Fu, Lai-Kuan Kong, Man Li

**Affiliations:** a School of Teacher Education, Heze University, Heze, China; b Faculty of Administrative Science and Policy Studies, Universiti Teknologi MARA (UiTM) Pahang Branch, Raub, Pahang, Malaysia; c Faculty of Business and Management, Universiti Teknologi MARA (UiTM) Pahang Branch, Raub, Pahang, Malaysia; d School of Marxism, Heze University, Heze, China

**Keywords:** Digital intelligence quotient, Scale development, Exploratory factor analysis, Confirmatory factor analysis, Primary school students

## Abstract

The development of a Digital Intelligence Quotient (DQ) scale for primary school students is the basis for research on the DQ of primary school students, which helps to scientifically diagnose the level and the current average DQ among Chinese primary school students. This study developed and validated a scale applicable to the assessment of DQ in Chinese primary school students where, the initial scale was first constructed; Then 1109 valid datasets were collected through purposive sampling and divided into Sample A and Sample B; Sample A was subjected to exploratory factor analysis and Sample B was tested by confirmatory factor analysis; The final validated scale consists of 22 items in 7 dimensions: digital identity, digital use, digital safety, digital security, digital emotional intelligence, digital literacy and digital rights. The scale has high reliability and validity and thus can be used as a reliable instrument for assessing DQ in Chinese primary school students.

## 1 Introduction

With the development of a new generation of digital technologies, the world has entered a digital era of intelligent guidance, cross-border integration and systematic innovation. Digitalization has profoundly changed our state of being, living, learning and working. Digital competence has become a core requirement for personal future and employment readiness [1]. Today's primary school students are digital citizens who have grown up with the internet and its various applications on hand. They are enjoying the convenience of the internet and at the same time undertaking the risks of its usage. In the digital age, the prevalence of undesirable information, cyber-addiction, privacy leakage and cyber-violence in the internet space has had an extremely serious negative impact on primary school students, whose mental development has not matured and capacity for discernment is generally weak [2]. According to a survey of children aged 8–12 years old from 29 different countries conducted by the Coalition for Digital Intelligence (CDI), more than half of the world's children are currently exposed to at least one online risk [[2], [3], [4]]. China's Research Report on the Risks of Youth Participation on Internet Platforms reveals that, relative to other age groups, the highest percentage of youth online risk cases is among those aged 13 years and younger [5]. It can be seen that primary school students, because of their young age, immature mental development and poor ability to distinguish between right and wrong, are more vulnerable to the harm of cyber risk events. In the face of the opportunities and challenges brought by the internet, a safe method by which Chinese primary school students can correctly utilize the internet, while avoiding the inherent hazards and fully enjoying its benefits has become an extremely urgent issue in the process of growth and education of the next generation in the digital environment.

The Digital Intelligence Quotient (DQ) is the mapping and development of information and digital literacy in the digital age [6]. DQ is a comprehensive digital competency rooted in universal moral values and is an essential skill for individuals to survive in the digital environment [7]. Possessing DQ enables primary school students to use, control and create technology wisely, to avoid and deal with cyber-risks that they may encounter, to better demonstrate competitiveness with the help of digital tools, and to adapt to the digital age [2]. Therefore, there is an urgent need for primary school students to possess a high DQ in the digital age, to control the digital world safely with critical thinking, to resist cyber-risks, and to form cyber-self-protection awareness and capability [4]. In order to improve DQ of primary school students, the current status and level of primary school students’ DQ should be established first. Consequently, it is urgent and important to develop a scientific and reasonable DQ scale for Chinese primary school students to scientifically diagnose their DQ levels.

In previous studies, it has been found that most of the research on DQ has been limited to discussing a DQ framework [6,8], and comparative studies with digital literacy and information literacy [9]. There has been relatively less research on the development of DQ scales. Na-Nan et al. developed a digital intelligence quotient scale with reference to the DQ framework [10]. However, this is a scale developed for corporate employees in Thailand and is not suitable for Chinese primary school students. How can a scientifically valid and reliable DQ scale be developed to assess the DQ levels of Chinese primary school students? Therefore, centred on this research question, this study develops a DQ scale suitable for Chinese primary school students in order to scientifically diagnose the dimensions of DQ.

## 2 Literature review

### 2.1 Connotations of the digital intelligence quotient

The first and second industrial revolutions shifted the value of human beings from manual labour to intellectual labour, highlighting the importance of knowledge, which led to the creation of the “intelligence quotient”, a measure of cognitive ability [11]. The third industrial revolution shifted the focus from knowledge to emotions and relationships, leading to the “emotional intelligence quotient” being developed as a soft skill for dealing with complex relationships [12]. Now, the Fourth Industrial Revolution has brought about artificial intelligence and automation, changing the nature of work and requiring us to develop higher-level competencies beyond traditional physical, cognitive, and soft skills, known as the “digital intelligence quotient” [8].

DQ was first introduced by Yuhyun Park, a Korean scholar and founder of the Digital Institute, at the 2016 World Economic Forum (WEF). She proposes that digital intelligence is a set of comprehensive technical, cognitive, metacognitive, and socio-emotional competencies, based on universal ethical values, that enable individuals to face the challenges of digital life and adapt to its demands [8]. It is a comprehensive digital competence rooted in universal ethical values that enables individuals to use, control and create technologies to meet the demands of digital life and contribute to the development of society [13]. DQ involves not only a deeper understanding and application of technology, but also to effectively utilize these technologies to enhance our cognitive abilities and improve our emotional intelligence performance. As a result, DQ has become a key competency for adapting to this changing era, combining the traditional intelligence quotient and emotional intelligence quotient with the modern technological environment to provide new dynamics for individuals to succeed in a digitalised world.

### 2.2 Frameworks of the digital intelligence quotient

**Fig. 1:**
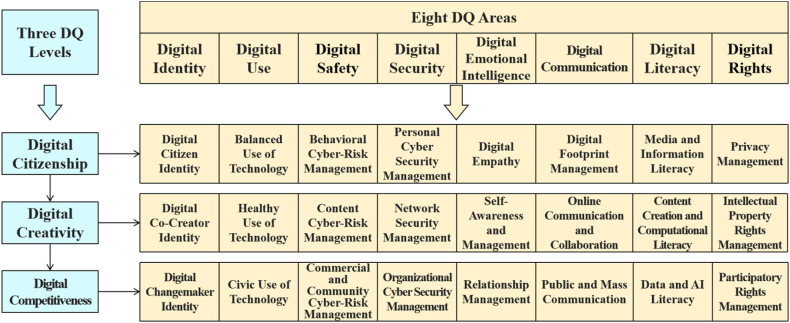
Global framework for DQ

In September 2018, WEF joined forces with the DQ Institute, the Organization for Economic Co-operation and Development, and the IEEE Standards Association to create CDI [8]. CDI aggregated more than 20 of the world's leading frameworks on DQ to summarize a global framework for DQ. There are three levels and eight domains that make up the Global Framework for DQ. The three levels of DQ are: digital citizenship, digital creativity, and digital competitiveness [8]. Digital citizenship is the ability to use digital technologies and media in a safe, responsible and ethical manner; Digital creativity is described as the ability to use digital technologies to accomplish creative activities, that is, the flexible ability to utilize digital tools or resources to create new knowledge, techniques, and solutions to problems; Digital competitiveness has been described as the ability to utilize digital technologies to create human well-being. Digital media and technologies are utilized to foster economic growth, positively impact social development, address global challenges in the digital economy, promote entrepreneurship and employment, and create new opportunities. This is the ultimate goal of the digital age. DQ is categorized into eight domains: digital identity (DI), digital use (DU), digital safety (DSA), digital security (DSE), digital emotional intelligence (DEI), digital communication (DC), digital literacy (DL), and digital rights (DR) [8]. Therefore, this creates an 8 x 3 matrix containing 24 capabilities, as shown in Fig. 1. DI refers to the ability to establish healthy offline and online identities; DU refers to the ability to use technology in a sensible and healthy way; DSA is the ability to understand, manage and avoid various cyber risks through the safe, responsible and ethical use of technology; DSE is the ability to detect, avoid and manage different levels of cyber threats to protect data, devices and systems; DEI is the ability of an individual to recognize, connect, and express emotions in the process of communicating with self or others in a digital life; DC is the ability to use technology to communicate and collaborate with others; DL is the ability to find, read, evaluate, synthesize, create, adapt and share information, media and technology; DR are the ability to understand and uphold human and legal rights when using technology.

In essence, the core purpose of the DQ framework is to facilitate the evolution of individuals from mere digital users to creators of digital content and competitors in the digital realm. The aim is to enable individuals not only to become technologically proficient, but also to go beyond the limitations of technology and utilize the power of the digital world to dominate and shape their lifestyles. These capabilities, once enhanced through individual efforts, enable individuals to maximize the benefits of digital technology in their lives and work, while minimizing digital and cyber risks [14].

### 2.3 Measures for the digital intelligence quotient

The DQ Global Framework released by CDI is currently used in most DQ measures. Na-Nan et al. [10] developed and validated a digital intelligence quotient scale for employees of small and medium-sized enterprises in Thailand with reference to the digital intelligence framework. The scale contains digital identity, digital use, digital safety, digital security, digital emotional intelligence, digital communication, digital literacy and digital rights, featuring 8 dimensions with 33 questions. Kulworatit et al. [15] developed the Internet Risk Assessment Scale utilizing the DQ framework and communication-based modeling. The scale contains digital identity, digital safety, digital emotional intelligence, digital rights, digital fear, digital greed, and digital unreasonable decision, featuring 7 dimensions with 27 questions. Saputra [16] tested the mediating role of DQ in leadership agility and employee engagement of the digital generation using the digital intelligence quotient scale developed by Na-Nan et al. [10]. Dusevic [17] explored the causal relationship between the Digital Economy and Society Index of EU countries and the DQ level of managers in EU direct centres, with a DQ self-assessment scale based on the European Framework for the Digital Competence of Educators: DigCompEdu [18]. Marnewick and Marnewick [6] identified the DQ competencies considered important by South African program managers from a societal perspective, which mainly include communicate and collaborate, digital rights, communication, and online identity. Zhao and Yang [19] constructed a DQ assessment framework for students in Chinese vocational colleges and universities, which is made up of three primary indicators, namely, digital information cognition, digital world survival, and digital society and culture, as well as 12 secondary indicators and 50 tertiary indicators.

In summary, DQ scales are currently designed primarily for corporate employees. Although Zhao and Yang [19] conducted a study on DQ for vocational students, the focus was on the measurement framework rather than a specific scale. It can be seen that research on assessing the DQ of student populations is still limited. As such, the current research results are still lacking in terms of assessing the DQ of Chinese primary school students. Therefore, this study aims to develop a scale that can measure the DQ level of Chinese primary school students to fill the gap in this area and to provide a more effective assessment basis for conducting digital literacy and training programmes.

## 3 Research methodology

### 3.1 Research design

This study followed Churchill's [20] scale development process and practical application of the DQ scale development by sequentially completing initial scale construction, data collection, exploratory factor analysis (EFA), and confirmatory factor analysis (CFA). In constructing the initial scale, this study collected and organized relevant items from the literature to form an item bank based on the DQ framework. The items were then streamlined and optimized through expert review. Feedback from the primary school students in the pre-test helped the researchers to adjust the scale to match the cognitive level of the primary school students, thus completing the development of the initial scale. In the data collection stage, the primary school students from grades four to six in Shandong Province were chosen as the respondents in this study. The study was conducted anonymously after obtaining the consent of schools and students. The researcher distributed the questionnaires through an online platform during the students' IT class periods, and the students filled in the questionnaires as required. After sorting and screening, the valid datasets were divided into sample A and sample B, which were used for the EFA and CFA, respectively. EFA was conducted on Sample A using SPSS 26.0 to determine the underlying structure of the DQ scale. Sample B was tested by CFA using SmartPLS 4.0 to assess the fit of the model, which in turn tested composite reliability, convergent validity, and discriminant validity to further justify the factor structure.

### 3.2 Initial scale construction

**Fig. 2:**
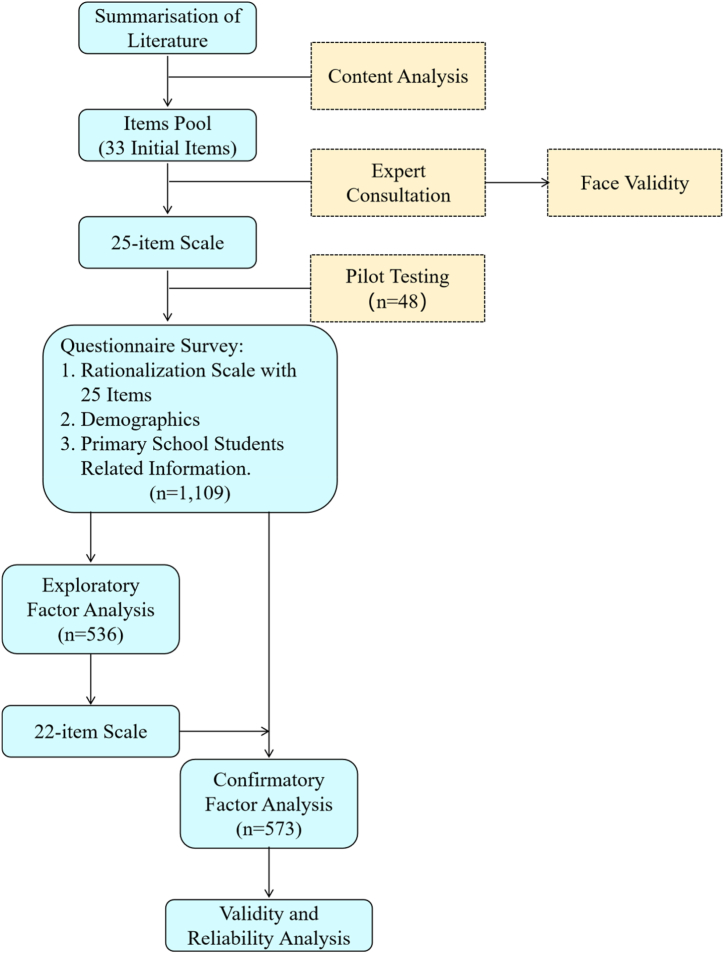
Scale development flowchart.

Based on the DQ framework proposed by Park and DQ Institute [8], this study, like Na-Nan et al.‘s [10], also adopts the DQ framework consisting of eight dimensions: DI, DU, DSA, DSE, DEI, DC, DL, and DR. The development of the DQ scale for primary school students in China was a methodical procedure that included multiple crucial stages, as shown in Fig. 2. At first, a comprehensive assessment of existing literature was carried out, which led to the identification of 33 possible items. Ensuring content validity was necessary in establishing a thorough foundation for the scale [21,22]. In order to verify the face validity, a panel of five experts was engaged to evaluate the relevance and comprehensibility of the items [23,24]. After incorporating their feedback, a total of eight questions were dropped, resulting in a more refined scale consisting of 25 items. The scale addresses 8 major dimensions: DI, DU, DSA, DSE, DEI, DC, DL, and DR.

Subsequently, a pilot study was undertaken with 48 primary school students to assess the reliability of these dimensions. The sample size of 48 students exceeded the suggested criterion of 30 respondents [25]. The results showed high internal consistency, with the following Cronbach's alpha values garnered: digital identity (0.861), digital use (0.843), digital safety (0.933), digital security (0.811), digital emotional intelligence (0.837), digital communication (0.874), digital literacy (0.938), and digital rights (0.869). All values exceeded the 0.70 criterion, indicating that the measurements are reliable. The steps undertaken ensure that the scale development process was rigorous, encompassing both content and face validity, as well as initial pilot reliability. These steps were followed by more in-depth statistical evaluations, such as EFA and CFA, to empirically confirm and establish the scale's validity and reliability [26,27].

### 3.3 Survey and participants

In the final cross-sectional research stage, this study selected the Shandong Province, which to date has made remarkable achievements in both digital economy and digital education, as the study area. The recruitment criteria for this study comprised of both inclusion and exclusion criteria. The inclusion criteria specified that the participants consist of both male and female students from fourth, fifth, and sixth grades. Students were chosen from urban and rural schools that have internet connectivity. In order to obtain a sample that accurately represents the population, students between the ages of 10 and 12 who attend school regularly and have a basic level of digital literacy were selected. Parental consent was acquired for every participant, and teacher approval was sought to assure school support. In addition, students were required to have engaged in digital education programs. In order to preserve the study's integrity, vulnerable populations, including kids with behavioural disorders, individuals who are mentally challenged or disabled, and those attending schools that lack internet access for students, were excluded. Additional grounds for exclusion encompass students diagnosed with certain learning challenges, students without parental consent, and those who do not have access to digital devices when they are at school. These criteria applied ensures that the sample accurately reflects the target demographic while also ensuring consistent exposure to digital environments. Through the meticulous definition of these inclusion and exclusion criteria, our objective is to establish a robust and reliable basis for the following phases of the DQ scale development.

The questionnaire was divided into two parts, the demographic information of the participants and the DQ scale scored via a 5 point Likert scale (1 = strongly disagree, 5 = strongly agree). The study used purposive sampling to select a number of classes in both urban and rural elementary schools. The researcher contacted the school and obtained permission from the parents/legal guardians to send the instructions for responding to the questionnaire and the QR code for the questionnaire to the teachers of the IT classes selected. During students' IT class periods, the classroom teachers and the researcher obtained students’ consent to distribute the questionnaires through the Questionnaire Star online platform. The student completes the questionnaire on the computer, where the student can withdraw from the survey at any time without any adverse effects. After data collection and screening, a total of 1109 valid datasets were collected.

In order to assess potential factor structure of the scale and the validity and reliability of the associated dimensions, the study divided the sample into two subsamples: Sample A and Sample B. Sample A contained 536 participants and sample B contained 573 participants. During the sample delineation process, the study ensured that the number of urban and rural classes in Sample A, was the same as in Sample B to ensure the fairness and accuracy of the study. Table 1 summarizes the demographic characteristics of Sample A and Sample B. The comparison reveals that the respondents in the two samples are largely similar in terms of gender, grade level, and geographic location of the school, reflecting the overall characteristics of the group.

**Table 1:** Demographic information of sample A and sample B.

Variable	Sample A (*N=536*)		Sample B (*N=573*)	
	Frequency	Percent	Frequency	Percent
Gender				
Male	279	52.05 %	300	52.36 %
Female	257	47.95 %	273	47.64 %
Grade				
Fourth Grade	191	35.63 %	202	35.25 %
Fifth Grade	179	33.40 %	196	34.21 %
Sixth Grade	166	30.97 %	175	30.54 %
Geographic Location				
City	255	47.57 %	278	48.52 %
Rural	281	52.43 %	295	51.48 %

### 3.4 Data analysis

This study is dedicated to finding the best model for scale rationalisation by implementing EFA and CFA. In order to explore the intrinsic structure of the DQ scale, SPSS 26.0 software was used in this study to conduct an EFA on sample A (N = 536). In order to measure the fit of the model, this study implemented a CFA on sample B (N = 573) using SmartPLS 4.0 software. On this basis, the composite reliability, convergent validity and discriminant validity of the scales were further assessed in this study to validate the reasonableness of the factor structure.

## 4 Analysis and results

### 4.1 Exploratory factor analysis

This study used SPSS 26.0 statistical software to conduct an EFA. The sample size of Sample A was 536, which exceeds the standard of at least 300 samples recommended by Tabachnick et al. [28]. Therefore, the sample size for Sample A is adequate and acceptable for subsequent analysis. Prior to factor analysis, in order to assess the appropriateness of the data, the Kaiser–Meyer–Olkin (KMO) test and Bartlett's test of Sphericity were conducted on Sample A in this study [29]. These tests are mainly applied to compare simple correlation coefficients and partial correlation coefficients between variables. When the KMO value is greater than 0.9 and the result of Bartlett's test of Sphericity is significant, this means that the sample is a good candidate for a principal component analysis [30]. The findings of the data garnered a KMO value of 0.927, which is above the 0.9 threshold, implying that the correlation of variables is robust and appropriate for factor analysis. Meanwhile, the Bartlett's test of Sphericity chi-square value was as high as 7125.804 with a degree of freedom of 300 and a significance level Sig. of 0.000 (p < .01), which also verifies that the sample data has a significant correlation structure. This suggests that the dataset from this study is statistically well suited for the next step, an EFA.

Subsequently, factors with eigenvalues greater than 1 were extracted using the SPSS 26.0 software correlation matrix principal component analysis module by adopting the maximum variance rotation method [31]. The rotation converged after 25 iterations, and the factor loading threshold was set to 0.50 for an EFA of the measurement items. Referring to previous studies [32], this study followed the following criteria to exclude inappropriate items. When the factor loading of an item after rotation was lower than 0.5, or the content of an item was clearly inconsistent with the theme of the factor to which it belonged, or an item had a high cross-loading (e.g., greater than 0.5) on more than one factor, such an item was removed [33]. Of course, this study will also choose to remove a factor if it contains fewer than three items [31]. Although the pilot study demonstrated that the DC construct was highly reliable, with a coefficient of 0.874, the EFA carried out using principal components analysis (with varimax rotation) revealed that the items DC1, DC2, and DC3 of the DC construct did not converge onto its own factor. Therefore, this study has decided to delete items DC1, DC2, and DC3, resulting in a 7-factor scale consisting of 22 items. The 7 factors extracted explained 72.865 % of the total variance, well exceeding the 60 % threshold [34]. The KMO value of the final scale was 0.911, which is greater than 0.9, and the Bartlett's test of Sphericity was significant (Approx. Chi-Square = 6065.742, df = 231, p = .000, <0.01). Table 2 shows the factor loading values, factor characteristics, and variance explained for the remaining items that had met the requirements set.

**Table 2:** The results of EFA.

Items	Component						
	1	2	3	4	5	6	7
DSA2: I don't add people I don't know online.	**0.835**	0.081	0.082	−0.002	0.110	0.094	0.121
DSA3: I will not put my school number, name, or other information online.	**0.828**	0.175	0.065	−0.025	0.007	0.221	0.092
DSA4: I do not view vulgar content or unhealthy websites.	**0.783**	0.139	0.057	0.044	0.142	0.319	0.070
DSA1: I don't like uploading photos or videos etc. of my daily life to the internet.	**0.770**	0.054	0.056	0.029	0.173	−0.063	0.177
DL1: I am able to search for information on the web.	0.217	**0.799**	0.150	0.183	0.147	0.168	0.062
DL2: I am able to select authentic and reliable information on the web.	0.114	**0.741**	0.194	0.159	0.341	0.103	0.135
DL3: I am able to use retrieved information wisely.	0.172	**0.717**	0.305	0.091	0.295	0.126	0.182
DEI3: I can express my attitude by liking or commenting on others' posts.	0.082	0.162	**0.837**	0.167	0.088	0.108	0.088
DEI1: I can express my feelings by posting on WeChat Moments or sharing a status update.	0.022	0.121	**0.739**	0.249	0.083	0.140	0.076
DEI2: I can feel the emotions of others from messages they post.	0.153	0.211	**0.731**	0.147	0.187	0.165	0.152
DI3: I use my real identity to chat with friends.	0.019	0.126	0.122	**0.819**	0.158	0.040	0.083
DI2: I use a real photo of myself in my profile.	0.005	0.012	0.145	**0.812**	0.064	0.029	0.120
DI1: I set nicknames when using QQ, WeChat, games and other software.	0.006	0.218	0.237	**0.688**	−0.066	0.104	0.039
DR3: When disseminating information, I am aware that I need to take some responsibility.	0.216	0.237	0.098	0.064	**0.753**	0.252	0.156
DR2: When I distribute information, I will consider whether it violates relevant laws and regulations.	0.234	0.296	0.150	0.076	**0.723**	0.310	0.089
DR1: When I use the work of others, I will indicate the source of information.	0.108	0.353	0.389	0.096	**0.563**	0.104	0.160
DSE1: I am able to use software or programs (e.g., mobile security guards, fraud prevention apps, etc.) to protect personal digital information.	0.228	0.074	0.158	0.118	0.308	**0.744**	0.189
DSE3: I am able to create complex passwords to secure my digital information.	0.171	0.268	0.291	0.078	0.169	**0.698**	0.107
DSE2: I do not respond to or communicate with people or things that threaten the security of my own digital information.	0.498	0.136	0.128	0.046	0.240	**0.566**	0.139
DU2: When I use digital media for learning, I don't use it for non-learning related things.	0.276	0.008	0.191	0.053	0.117	0.006	**0.781**
DU3: I am able to allocate my time wisely when using digital media.	0.188	0.146	0.064	0.094	0.292	0.200	**0.732**
DU1: I often use digital media for online learning.	0.028	0.374	0.099	0.282	−0.103	0.253	**0.610**
Eigenvalue	3.264	2.435	2.404	2.118	2.069	1.925	1.816
Variance explained (%)	14.836	11.069	10.926	9.625	9.403	8.752	8.255
Cumulative variance explained (%)	14.836	25.905	36.830	46.456	55.858	64.610	72.865
Cronbach's alpha	0.866	0.864	0.804	0.741	0.818	0.798	0.706

Notes: Extraction Method: Principal Component Analysis.

Rotation Method: Varimax with Kaiser Normalization.

Factor 1 contained four items, DSA1, DSA2, DSA3, and DSA4. Factor 1 was identified as DSA with a contribution of 14.836 %. Factor 2 contained three items, DL1, DL2, and DL3. Factor 2 was identified as DL with a contribution of 11.069 %. Factor 3 contained three items, DEI1, DEI2, and DEI3. Factor 3 was identified as DEI with a contribution rate of 10.926 %. Factor 4 contained three items, DI1, DI2, and DI3. Factor 4 was identified as DI with a contribution of 9.625 %. Factor 5 contained three items, DR1, DR2, and DR3. Factor 5 was identified as DR with a contribution of 9.403 %. Factor 6 contained three items, DSE1, DSE2, and DSE3. Factor 6 was identified as DSE with a contribution of 8.752 %. Factor 7 contained three items, DU1, DU2, and DU3. Factor 7 was identified as DU with a contribution of 8.255 %.

After confirming the factor structure of the scale, the 7 factors were analyzed for reliability in this study in order to verify the internal consistency and stability of the scale. The Cronbach's alpha coefficients for each factor, which is one of the indicators commonly used to assess the reliability of a scale, were calculated using SPSS 26.0 software, and the results are detailed in Table 2. It can be seen that the Cronbach's α values for all factors lie in the range of 0.706–0.866, with all exceeding the threshold of 0.7 [35]. This indicates that the internal consistency of the scale is high and that the internal structure of the factors is stable and reliable while meeting statistical requirements.

### 4.2 Confirmatory factor analysis

**Fig. 3:**
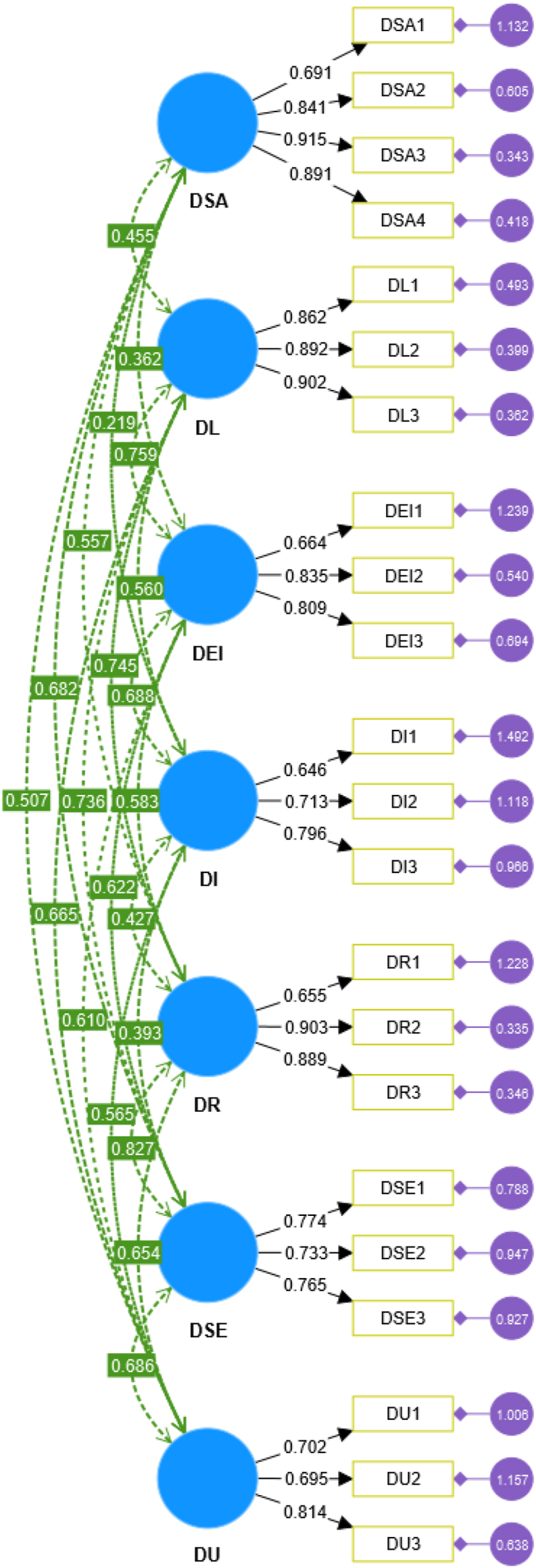
Dq CFA model.

Although EFA was able to provide an initial delineation of the factor structure of the construct, it did not determine the overall fit of the factor structure [36]. Therefore, this study employed SmartPLS 4.0 software to conduct a CFA of sample B (N = 573) using a covariance-based structural equation modeling analysis method. The overall factor structure fit was examined in this study, and the model fit was assessed using model fit metrics. The CFA model is shown in Fig. 3.

Referring to the suggestion of Hair et al. [34], the relevant fit metrics were selected to judge the fit of the model, which were mainly examined in terms of absolute fit metrics and relative fit metrics [37]. A smaller value of ChiSqr/df indicates a better fit between the covariance matrix of the theoretical model and the actual data [38]. Generally, ChiSqr/df values between 2.0 and 5.0 are acceptable [37,39]. The value of root mean square error of approximation (RMSEA) is between 0.05 and 0.08, which represents a good fit of the model [40]. The goodness of fit index (GFI) and adjusted goodness of fit index (AGFI) values should be greater than 0.90, with values closer to 0.90 being acceptable [37,41]. The Standardized root mean square residual (SRMR) is usually less than 0.08 [37]. The relative fit metrics normed fit index (NFI), TuckerLewis index (TLI) and comparative fit index (CFI) values should be greater than 0.90 [34,42]. Table 3 shows the garnered values, Chi-square = 589.784, Degrees of freedom = 188.000, P value = 0.000, ChiSqr/df = 3.137, RMSEA = 0.061, SRMR = 0.053, GFI = 0.912, AGFI = 0.881, NFI = 0.928, TLI = 0.938, CFI = 0.950. The model fitting results all met the specified thresholds and the model fit was deemed good.

**Table 3:** The results of CFA.

Fitness Index	Statistical Results	Fitting Criteria
Chi-square	589.784	
Degrees of freedom	188.000	
P value	0.000	<0.01
ChiSqr/df	3.137	2.0–5.0
RMSEA	0.061	0.05–0.08
RMSEA LOW 90 % CI	0.056	
RMSEA HIGH 90 % CI	0.067	
SRMR	0.053	＜ 0.08
GFI	0.912	＞ 0.90
AGFI	0.881	
NFI	0.928	
TLI	0.938	
CFI	0.950	

### 4.3 Reliability and validity analysis

This study then continued with an in-depth analysis of the composite reliability (CR), convergent validity, and discriminant validity of the scales. Usually, when the value of CR exceeds 0.7, it is considered acceptable [43]. According to the data in Table 4, the CR of the 7 factors ranged from 0.764 to 0.916, all exceeding 0.7, which indicates that the scale performed strongly in terms of reliability. In other words, the items in the scale show consistency and stability in measuring the same factor. In general, convergent validity needs to fulfil three main criteria: first, the standardized factor loadings of all items should exceed 0.6 [44]; second, the average variance extracted (AVE) should be larger than 0.5 [44]; and lastly, the CR needs to surpass 0.7 [43]. When these three conditions are satisfied simultaneously, the sample has good convergent validity. As can be seen from the data in Table 4, all the items have factor loadings between 0.646 and 0.915, which were higher than the threshold of 0.6; the CR of each factor also exceeded the criterion of 0.7, as well as the AVE values which were all higher than 0.5. Therefore, this study confirms that the scale performed excellently in terms of convergent validity and displayed good convergence between the data.

**Table 4:** Convergent validity and reliability assessment.

Factor	Items	Factor loading	CR	AVE
Factor I DSA	DSA1	0.691	0.903	0.704
	DSA2	0.841		
	DSA3	0.915		
	DSA4	0.891		
Factor II DL	DL1	0.862	0.916	0.784
	DL2	0.892		
	DL3	0.902		
Factor III DEI	DEI1	0.664	0.810	0.598
	DEI2	0.835		
	DEI3	0.809		
Factor IV DI	DI1	0.646	0.764	0.520
	DI2	0.713		
	DI3	0.796		
Factor V DR	DR1	0.655	0.852	0.678
	DR2	0.903		
	DR3	0.889		
Factor VI DSE	DSE1	0.774	0.801	0.574
	DSE2	0.733		
	DSE3	0.765		
Factor VII DU	DU1	0.702	0.780	0.546
	DU2	0.695		
	DU3	0.814		

This study assessed discriminant validity utilizing the Heterotrait-Monotrait (HTMT) correlation ratio criterion to ensure differentiation between the factors. According to Franke and Sarstedt [45] and Henseler et al. [46], discriminant validity is established when the HTMT result does not exceed 0.9. Table 5 indicates that all HTMT values ranged from 0.231 to 0.873, which were less than 0.9. This is ample evidence that the scale excelled in terms of discriminant validity, with clear differentiation between the factors, thus fulfilling the study's requirements for discriminant validity. These approaches of evaluation have been utilized in the research that was carried out by Li et al. [47,48].

**Table 5:** Discriminant validity assessment (HTMT).

Factor	DEI	DI	DL	DR	DSA	DSE	DU
DEI							
DI	0.737						
DL	0.764	0.576					
DR	0.659	0.500	0.826				
DSA	0.373	0.231	0.473	0.578			
DSE	0.622	0.402	0.738	0.873	0.693		
DU	0.614	0.613	0.672	0.720	0.535	0.689	

## 5 Discussion

In the digital era, DQ has become an indispensable basic competency yardstick for individuals. Enhancing the DQ of primary school students can help them better adapt to digital learning and improve their ability to recognize and prevent cyber risks. Therefore, the development of a scientific DQ assessment scale is crucial for the digital education of primary school students. The contribution of this study is the development and validation of a DQ scale for primary school students applicable to the Chinese context. Using data from 1109 Chinese primary school students, through EFA and CFA, this study constructed a scale with seven dimensions – DI, DS, DSA, DSE, DEI, DL, and DR – featuring a total of 22 items on the DQ scale, aiming to comprehensively assess the digital competence of Chinese primary school students.

This study found that the structure of DQ scale matched the empirical data from Chinese primary school students, and that the 22 question items contained in the scale were successfully classified into seven dimensions. This result is somewhat different from the DQ assessment models proposed by Park & DQ Institute [8], Na-Nan et al. [10], and Kulworatit et al. [15]. In comparison with the Internet Risk Assessment Scale developed by Kulworatit et al. [15], we found that a total of 4 dimensions were the same, namely digital identity, digital safety, digital emotional intelligence, and digital rights, while the other 3 dimensions are different. The DQ model proposed by Park & DQ Institute [8] and Na-Nan et al. [10] contains 8 dimensions, specifically DI, DU, DSA, DSE, DEI, DC, DL, and DR. The DQ scale for Chinese primary school students developed in this study corresponds to 7 of the 8 dimensions, but does not form the dimension of DC.

The EFA carried out determined that the components DC1, DC2, and DC3 were insufficient to establish independent digital communication dimensions. One possible reason for this result is that children between the ages of 10 and 12, who attend primary school, are still closely monitored by their parents when it comes to their online activities. During this period of development, children's cognitive capacities are still developing, which means they have limited ability to independently navigate and use digital communication in an effective and safe manner [49]. Parents have a vital role in managing their children's online activities to protect them from any dangers and ensuring they behave appropriately [50]. Therefore, the regulated setting in which these students function may hide the inherent grouping of digital communication skills into separate categories, as indicated by the EFA results.

## 6 Implication and limitations

### 6.1 Theoretical implication

The theoretical implication of this study are significant in the following two areas:

First, this study provides important support for promoting the construction of a disciplinary framework for DQ in China. Based on the conceptualization and global framework of DQ, this study developed a DQ scale for primary school students that is applicable for the Chinese context. The findings of this study help to further deepen and enrich the theoretical research related to DQ in China. It lays a solid theoretical foundation for future research on DQ education in China and helps to promote the theoretical development and practical exploration of DQ education in China.

Second, this study helps to deepen the research on the variability of DQ in China. By developing the DQ scale for Chinese primary school students, it is possible to assess the developmental level of DQ among primary school students and to understand the current status and level of DQ among Chinese primary school students. The results of the assessment not only help to scientifically diagnose the primary school students’ level of DQ, but also to update the data of DQ studies of primary school students in the digital era. This will further expand the theoretical research on the developmental level and variability of DQ among primary school students, and provide scientific references for designing more precise educational strategies and teaching programmes.

### 6.2 Practical implication

The practical implication of this study are significant in the following two ways:

First, this study offers a new measuring instrument for scientifically assessing the DQ level of Chinese primary school students. The development of the DQ scale for primary school students can provide a DQ assessment tool for educational administrations and educators. This helps them to scientifically evaluate the overall Chinese primary school students’ development level of DQ and quantitatively reflect the effectiveness and shortcomings of the same, so as to provide data support for the formulation of more targeted education policies and teaching schemes.

Second, this study helps to scientifically construct a DQ curriculum system suitable for Chinese primary school students. Educational administrations and educators can scientifically analyze the DQ related thinking styles and behavioural tendencies of primary school students through an in-depth understanding of the index system of the DQ and its corresponding behavioural manifestations. Based on these analyses, they can develop a more targeted curriculum system for fostering DQ of primary school students and a comprehensive enhancement program to help students apply their cognitive abilities to overcome digital challenges, thereby improving their learning effectiveness, especially their problem-solving skills in the online education environment.

### 6.3 Limitations and future research

The limitations of this study are mainly in two areas:

First, there were some geographical limitations in the selection of the sample, as the sample was mainly from the Shandong Province of China. While this provides valuable data support for the study, future research might collect data from a larger area and possibly other nations to test the DQ scale's generalizability for primary school pupils.

Second, although the dimension DC was included in the initial scale, it could not be established as a separate factor in the subsequent EFA, and thus was not included in the final scale. However, with the continuous development and popularization of digital technology, the use of digital technology for communication and collaboration has become an important means of digital learning for primary school students. Therefore, in future studies, DC should be considered to be recognized and included as a separate dimension in order to assess the DQ of primary school students in a more comprehensive way.

## 7 Conclusion

This study developed and validated a DQ scale for primary school students suitable for the Chinese context. The scale was demonstrated to have satisfactory reliability and validity through EFA and CFA carried out on datasets from 1109 Chinese primary school students. The findings of the study are summarised as follows: (1) The scale consists of 22 items divided into 7 dimensions: digital identity, digital use, digital safety, digital security, digital emotional intelligence, digital literacy and digital rights, all cumulatively explaining 72.865 % of the variance rate. (2) The internal consistency coefficients for 7 dimensions of the scale ranged from 0.706 to 0.866, and CR ranged from 0.764 to 0.916, showing high reliability. (3) The factor loadings of all the items of the scale ranged from 0.646 to 0.915, with AVE greater than 0.5, and the HTMT values ranged from 0.231 to 0.873, indicating that the scale has acceptable convergent and discriminant validity. The results of the factor analyses indicates that the scale can be used to test the DQ of Chinese primary school students.

## Funding

This work was supported by Shandong Educational Science Planning Program (2021ZC011).

Li Jing was funded by the teachers' visiting training grant for ordinary undergraduate colleges and universities in Shandong Province, China.

## Ethics approval

The studies involving human participants were reviewed and approved by Heze University Ethics Committee.

## Informed consent

Informed consent was obtained from all individual participants included in the study.

## Data availability statement

The datasets generated for this study are available on request to the corresponding author.

## CRediT authorship contribution statement

**Jing Li:** Writing – original draft, Formal analysis, Conceptualization. **Soon-Yew Ju:** Supervision, Formal analysis. **Caixia Zhu:** Methodology, Investigation. **Ying Yuan:** Methodology, Investigation. **Min Fu:** Investigation. **Lai-Kuan Kong:** Writing – review & editing, Supervision. **Man Li:** Writing – review & editing, Writing – original draft.

## Declaration of competing interest

The authors declare that they have no known competing financial interests or personal relationships that could have appeared to influence the work reported in this paper.
